# Circular RNA DOCK1 promotes bladder carcinoma progression via modulating circDOCK1/hsa‐miR‐132‐3p/Sox5 signalling pathway

**DOI:** 10.1111/cpr.12614

**Published:** 2019-04-14

**Authors:** Peihua Liu, Xiaozhou Li, Xi Guo, Jinbo Chen, Chao Li, Minfeng Chen, Longfei Liu, Xiangyang Zhang, Xiongbing Zu

**Affiliations:** ^1^ Department of Urology, Xiangya Hospital Central South University Changsha China; ^2^ Department of Urology, Hunan Provincial People's Hospital the First Affiliated Hospital of Hunan Normal University Changsha China

**Keywords:** bladder carcinoma, ceRNA, circDOCK1/hsa‐miR‐132‐3p/Sox5, circular RNA

## Abstract

**Objectives:**

To reveal the role of circular RNA (circRNA) DOCK1 (circDOCK1) as a potential biomarker and therapeutic target and its competing endogenous RNA mechanism in bladder carcinoma (BC).

**Methods:**

The next‐generation sequencing (NGS) technology was introduced to screen the circRNA expression profiles of BC using microarray. qPCR and Western blots assay were employed to measure the gene expression in different groups. Cell counting kit‐8, EdU and transwell assays were applied to detect the cell viability, proliferation and migration potential, respectively. Luciferase reporter assay was used to test the binds between hsa‐miR‐132‐3p/Sox5. Xenografted tumour growth of nude mice was performed to test the role of circDOCK1 in vivo.

**Results:**

CircDOCK1 was upregulated in BC tissues and cell lines. Repression of circDOCK1 reduced cell viability, inhibited cell proliferation and curbed the cell migration potential of BC cell. CircDOCK1 played its role via regulation of circDOCK1/hsa‐miR‐132‐3p/Sox5 pathway in BC cells. Suppression circDOCK1 inhibited the tumour growth in vivo.

**Conclusion:**

In this study, we revealed that circDOCK1 affected the progression of BC via modulation of circDOCK1/hsa‐miR‐132‐3p/Sox5 pathway both in vitro and in vivo and providing a potential biomarker and therapeutic targets for BC.

## 1 INTRODUCTION

Bladder carcinoma (BC) is one of the most commonly diagnosed urologic carcinomas, with distinctive morbidity and mortality, and is the seventh most prevalent carcinoma in males worldwide.^1^ Statistics indicate that approximately 440 000 new cases of BC are diagnosed, and approximately 130 000 BC patients die worldwide every year.^1^ Approximately 75% of BC patients with high‐risk, non‐muscle‐invasive BC suffer from the high recurrence rate and poor prognosis and, even worse, die within 10 years after diagnosis.^2^ Although non‐muscle‐invasive BC is more common, the disease has a high risk of progressing to muscle‐invasive BC.^3^ Currently, there is no effective therapy beyond surgery, chemotherapy and radiation for BC.^4^, ^5^ Thus, interventions to control progression and metastasis are critical for the treatment of BC, but the molecular mechanisms underlying the development, metastasis and progression of BC are still largely unclear.

In recent decades, an increasing number of circular RNAs (circRNAs) have been discovered.^6^ CircRNAs are a newly emerging class of evolutionarily conserved endogenous cellular RNAs are characterized by covalently closed continuous loops without polarity or a polyadenylated tail.^7^ CircRNAs are formed from precursor mRNAs by back‐splicing, resulting in circularization. Increasing evidence indicates that circRNAs play important roles in various cellular processes, including chromatin remodelling, cell proliferation, cell cycle regulation, apoptosis, migration and invasion, adhesion, differentiation and carcinogenesis.^8^, ^9^

Increasing evidence has demonstrated that circRNAs are widely involved in carcinogenesis and the malignant behaviour of human carcinomas, such as breast carcinoma, hepatocellular carcinomas, pancreatic carcinoma, gastric carcinoma, ovarian carcinoma, renal carcinoma, lung carcinoma, colon carcinoma and BC.^11^, ^12^ Furthermore, circRNAs could be biomarkers for diagnosis and therapeutic targets in BC.^15^, ^16^ Previous work showed that circ‐ITCH could act as a tumour suppressor by forming a novel signalling axis involving circ‐ITCH/miR‐17, miR‐224/p21 and PTEN, which might provide a potential biomarker and therapeutic target for BC.^13^ Despite the increasing understanding of the role of circRNAs in carcinoma, research into the activity and underlying mechanisms of circRNAs may lead to significant advances in the diagnosis and treatment of BC.

However, to date, the detailed molecular mechanisms underlying the role by which circRNAs exert their function in BC have not been well elaborated. In recent years, the regulatory mechanism described in the competing endogenous RNA (ceRNA) hypothesis has attracted increasing attention from scientists. The ceRNA hypothesis states that long non‐coding RNAs (lncRNAs), mRNAs and pseudogene transcripts could act as molecular sponges that compete with mRNA for binding to microRNA response elements (MREs), resulting in the repression of the activities of microRNAs (miRNAs), thus restoring the expression and activities of mRNAs.^17^, ^18^ Given that some circRNAs are derived from the same exon of an mRNA, circRNA was proposed as a novel member of the ceRNA family.^19^ Previous studies documented that circRNAs could play a role in the development and progression of carcinomas such as hepatocellular carcinoma, lung carcinoma, colon carcinoma, breast carcinoma, gastric carcinoma and BC via ceRNA regulatory mechanisms.^20^, ^21^ Zhenyu Zhong and colleagues revealed that circRNA‐MYLK acted as a ceRNA for miR‐29a to affect epithelial‐mesenchymal transition (EMT) and the development of BC by activating VEGFA/VEGFR2 and the downstream Ras/ERK signalling pathway.^22^ However, the role and underlying mechanisms of circRNAs in the tumorigenesis, development and progression of BC remain largely unclear.

Thus, we aimed to examine the role and underlying mechanism of circRNAs in BC. We systematically identified circRNA expression profiles in BC tissues using a microarray and identified the increased expression of circDOCK1 in BC tissues and cell lines. Further study revealed that circDOCK1 affected the progression of BC via the modulation of the circDOCK1/hsa‐miR‐132‐3p/Sox5 pathway both in vitro and in vivo. Thus, although the role and underlying molecular mechanisms of circRNAs remain largely indeterminate, our study showed the role of the circDOCK1/hsa‐miR‐132‐3p/Sox5 pathway in BC and indicated a new therapeutic approach for BC.

## 2 MATERIALS AND METHODS

### 2.1 Human specimens

This study was approved and supervised by the Research Ethics Committee of the Xiangya Hospital, Central South University. Totally 23 BC tissue specimens and 32 normal bladder tissue specimens were collected from patients undergoing surgical resection at Xiangya Hospital, Central South University. The pathological diagnosis was in accordance with the tumour node metastasis classification for stage (2002 International Union Against Carcinoma) and histopathologic grade (WHO 1973). Fresh samples were pathologically confirmed using standard haematoxylin staining and immediately snap‐frozen in liquid nitrogen and stored at −80°C until use. Written informed consent agreements were obtained from all patients for research purposes.

### 2.2 Cell culture

Immortalized human bladder epithelial SVHUC‐1 cells and the bladder carcinoma (BC) cell lines BIU‐87, EJ‐m3, T24 and 5673 were obtained from our own laboratory. Cell lines were grown in media as recommended by the American Type Culture Collection (ATCC). Cells were routinely cultured in Dulbecco's modified Eagle's medium (DMEM; Invitrogen, Carlsbad, CA) supplemented with 10% foetal bovine serum (FBS), 100 μg/mL penicillin, 0.1 mg/mL streptomycin and 2 mmol/L l‐glutamine. Cells were maintained at 37°C in an incubation cabinet containing 5% CO_2_.

### 2.3 RNA interference

The stealth RNA interference (RNAi) oligonucleotides specifically targeting hsa_circ_0020394 and Sox5 were designed and synthesized by GenePharma (Shanghai, China); the sequences were as follows: hsa_circ_0020394‐siRNA1, 5′‐CCGGGUGAAGCUUUUUAUATT‐3′ and hsa_circ_0020394‐siRNA2, 5′‐GUGAAGCUUUUUAUAACUATT‐3′; Sox5‐siRNA1, 5′‐GCAGCAACAAGAACAAATT‐3′, Sox5‐siRNA2, 5′‐GCCATTAATGATTCCCGTA‐3′ and Sox5‐siRNA3, 5′‐GCCATATTATGAGGAGCAA‐3′; hsa‐miR‐132‐3p mimic, 5′‐UAACAGUCUACAGCCAUGGUCG‐3′; and hsa‐miR‐132‐3p inhibitor, 5′‐CGACCAUGGCUGUAGACUGUUA‐3′.

### 2.4 RNA extraction, RT‐PCR and qRT‐PCR

Total RNA was isolated from bladder carcinoma (BC) and normal tissues and cells using TRIzol reagent (Invitrogen) according to the manufacturer's protocol. Subsequently, total RNA was quantified using a NanoDrop ND‐1000 spectrophotometer (Thermo Scientific). Then, 1 µg of RNA was treated with RNase‐free DNase and used for reverse transcription with a cDNA Synthesis SuperMix kit (Donghuan Biotech, Shanghai, China). qRT‐PCR was performed with a SYBR Green array (Donghuan Biotech) and an ABI PRISM 7500 Fast Real‐Time PCR System (Applied Biosystems). The data were analysed by using the 2^−ΔΔCt^ method. The primers used for qPCR were synthesized at Sango Biotech (Shanghai, China). The relative expression of the different genes was normalized to β‐actin expression, and arbitrary units were employed to display the normalized gene expression. The primers used in this study are listed in Table 1.

**Table 1:** Primers for real‐time PCR

Primers	Sequences (5′‐3′)
hsa_circ_0020394‐F	GCTCTTCAGGTCCATCAATGAC
hsa_circ_0020394‐R	CGATCTGTAAAGAAAGTTCATCCG
hsa_circ_0087856‐F	GCAACTACGACAGCAACAAC
hsa_circ_0087856‐R	CCTGCTACTGGAAAGGCATC
hsa_circ_0021714‐F	ATCCGGAAGTGCTACTTCAG
hsa_circ_0021714‐R	GGGAACCTGGAGTGTCGC
hsa_circ_0026358‐F	TATGAGGAGATGGCCAAATGC
hsa_circ_0026358‐R	CTGCAGCAGCGTCCAC
hsa_circ_0044097‐F	GACTCACACCCAGGGACAAG
hsa_circ_0044097‐R	CCTCATTCTCTATGTCCTCTGC
hsa_circ_0000629‐F	GTGGATGACAACACAGTTATGCG
hsa_circ_0000629‐R	CCTTTCCATTAGCTTCTCTTTATCCC
hsa_circ_0001226‐F	AGGGTGGCGATCTGCTTC
hsa_circ_0001226‐R	GAAACTGCTGAGGAGGTGAAG
hsa_circ_0103730‐F	CCTTCAAGAAGGGCATACACAG
hsa_circ_0103730‐R	TGGTGTGGCCTATCTGCAG
hsa‐miR‐132‐3p‐F	GCAACGTAACAGTCTACAGCC
hsa‐miR‐132‐3p‐R	CCAGTGCAGGGTCCGAGGTA
hsa‐miR‐132‐3p‐stem‐loop RT primer	GTCGTATCCAGTGCAGGGTCCGAGGTA TTCGCACTGGATACGACCGACCAT
GAPDH‐F	GGGAAACTGTGGCGTGAT
GAPDH‐R	GGGAAACTGTGGCGTGAT
Sox5‐F	AGGTTTGGACTCACTTGACAGG
Sox5‐R	GTGAGGCTTGTTGGGAAAACTC

### 2.5 Western blot analysis

The samples were lysed using radioimmunoprecipitation assay (RIPA) lysis buffer, and protein quality was assessed using a BC detection kit (Donghuan Biotech, Shanghai, China). Then, the samples were separated via 12% sodium dodecyl sulphate‐polyacrylamide gel electrophoresis (SDS‐PAGE) and electro‐transferred onto a polyvinylidene difluoride membrane. After incubation with 5% milk for 1 hour, the membranes were incubated with the various primary antibodies at 4°C overnight. The next day, the membranes were incubated with secondary antibody for approximately 1 hour at room temperature. Blots were visualized with an enhanced chemiluminescence detection kit (Donghuan Biotech). The primary and secondary antibodies used in this study were anti‐SOX5 (Abcam, UK; 1:2000 dilution) and anti‐GAPDH primary antibodies (Donghuan Biotech, 1:5000 dilution) and a fluorescent goat anti‐rabbit secondary antibody (Abcam, UK; 1:4000 dilution).

### 2.6 Cell counting kit‐8 assay

A cell counting kit‐8 (CCK8; Donghuan Biotech) assay was employed to measure cell viability, according to the manufacturer's instructions. Cells exposed to different treatments were seeded in a 96‐well plate, and the CCK8 reagents were added after 24 hour. Then, the absorbance was read at 450 nm using a microplate reader after 2 hour of incubation with the CCK8 reagents. Finally, the data were collected and analysed, and arbitrary units were used to show the normalized relative differences among different groups.

### 2.7 5‐Ethynyl‐2′‐deoxyuridine assay

Cell proliferation was examined by an 5‐ethynyl‐2′‐deoxyuridine (EdU) incorporation assay (Donghuan Biotech) according to the manufacturer's protocol. In brief, cells exposed to different treatments were plated in 48‐well plates at a density of 2.5 × 10^4^ cells/well. After different treatments, the cells were incubated with 10 mmol/L EdU for 2 hour at 37°C, fixed with 4% paraformaldehyde for 30 minute at room temperature, permeabilized with 0.3% Triton X‐100 and then stained with Apollo Staining reaction liquid followed by Hoechst for 15 minute at room temperature. The cells were observed, imaged and counted in three random fields (×10) using a fluorescence microscope (ZKX53; Olympus, Tokyo, Japan).

### 2.8 Cell migration assay

The migration potential of the cells was measured using transwell chambers purchased from Corning (Corning). In brief, 5.0 × 10^4^ cells exposed to different treatments were plated in the top chamber, which contained medium supplemented with 0.1% FBS, while the lower chamber was filled with medium containing 10% FBS. After incubation for 24 hour at 37°C, cells adhering to the upper chamber were scraped by a cotton swab, stained with crystal violet for 15 minute at room temperature and air‐dried for approximately 30 minute at room temperature. The cells were observed, imaged and counted in three random fields (×10) using a fluorescence microscope (ZKX53; Olympus).

### 2.9 Luciferase reporter assay

The Sox5 3′‐UTR sequences were amplified by PCR and cloned into the XhoI/NotI restriction sites of the psiCHECK‐2 luciferase reporter plasmid (Promega). The sequence of the putative binding site (hsa‐miR‐132‐3p) was substituted as indicated by Sox5 mutation. For the luciferase assay, 293T cells were seeded into 6‐well plates (4.5 ×  10^5^ cells/well) and co‐transfected with the Sox5 Wt or Sox5 Mut 3′‐UTR reporter plasmids (2.5 μg) and the hsa‐miR‐132‐3p mimic (50 nmol/L) or miR‐Ctrl (50 nmol/L) using Lipofectamine 3000 reagent (Invitrogen) according to the manufacturer's instructions. Renilla luciferase activities were examined 48 hour after transfection using a Dual‐Luciferase Assay System (Promega).

### 2.10 Fluorescence in situ hybridization

In brief, cells exposed to different treatments were plated in 48‐well plates at a density of 2.5 × 10^4^ cells/well. Next day, the cells were hybridized in hybridization buffer (Servicebio Technology, Wuhan, China) with digoxin (Dig)‐ and biotin (Bio)‐labelled single‐stranded DNA probes at 37°C overnight. Then, the digoxin‐labelled probes (5′‐DIG‐ATAGT TATAA AAAGC TTCAC CCGGA CGG‐DIG‐3′) specific to has‐circ‐0020394 back‐splice region were added (Servicebio Technology), following with Cy3‐conjugated anti‐digoxin and FITC‐conjugated anti‐biotin antibodies (Jackson ImmunoResearch Inc, West Grove, PA). In addition, the sell nuclei were stained with Hoechst. At last, the results were obtained on a fluorescence microscope (ZKX53; Olympus).

### 2.11 Statistical analysis

All data are expressed as the mean ± SEM All experiments were performed at least three times. The data were analysed by SPSS statistical software (version 13.0.0). Statistical analysis was performed using Student's *t* test or one‐way ANOVA. *P < 0.05 was considered statistically significant, and **P < 0.01 was considered highly statistically significant in a two‐tailed Student's *t* test.

## 3 RESULTS

### 3.1 CircDOCK1 (hsa_circ_0020394) was significantly upregulated in BC

**Figure 1:**
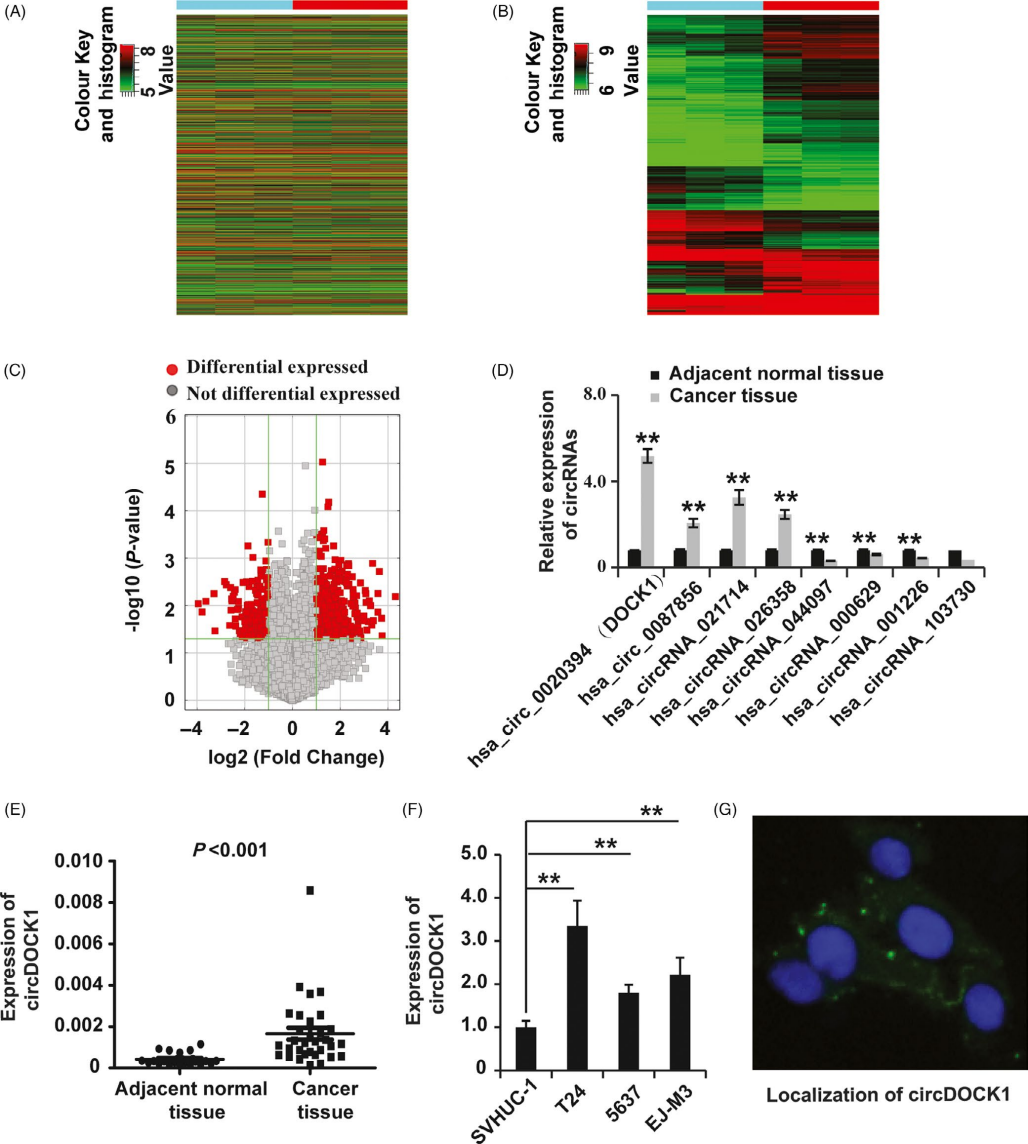
Identification of differentially expressed circRNAs in BC. (A and B) Hierarchical clustering of the circRNA expression data shows distinguishable gene expression profiles among the BC and adjacent normal tissues. (C) Volcano plots for circRNAs differentially expressed between the two different tissue types. (D) qPCR analysis of circRNAs differentially expressed between the two different tissue types. GAPDH was used as the control. (E) qPCR analysis of circDOCK1 expression in BC (n = 23) and adjacent normal tissues (n = 32). GAPDH was used as the control. (F) qPCR analysis of circDOCK1 expression in immortalized human bladder epithelial SVHUC‐1 cells and the BC cell lines BIU‐87, EJ‐M3, T24 and 5673. (G) Localization of circDOCK1 detected by fluorescence in situ hybridization in EJ‐M3 cells. Cell nuclei were counterstained with Hoechst (blue). GAPDH was used as the control. Each experiment was repeated a minimum of three times. The symbol * denotes a significant difference (*P* < 0.05), while ** represents a highly significant difference (*P* < 0.01) in a two‐tailed Student's *t* test

To examine the role of circRNAs in BC, the circRNA and mRNA expression profiles in 3 BC tissues and 3 matched normal bladder tissues were investigated using microarray analysis. The results demonstrated that thousands of circRNA transcripts were differentially expressed between BC and normal tissues (Figure 1A). Of these differentially expressed circRNAs, 734 showed significant differential expression (fold change >2.0 and *P* < 0.05), including 478 upregulated and 256 downregulated circRNAs in BC tissues (Figure 1B). Then, the significantly differentially expressed circRNAs were subjected to fold change filtering and the volcano plot filtering analysis displayed significantly differentially expressed circRNAs in BC and normal tissues (Figure 1C).

To confirm the results from the microarray, we examined the expression of eight circRNAs selected from the array results by qRT‐PCR assay. The results showed that circDOCK1 was the circRNA with the greatest increase in expression in the carcinoma tissues (Figure 1D). To elucidate the possible role of circDOCK1 in BC, we collected 32 non‐tumour tissues and 23 BC tissues and employed qRT‐PCR to examine circDOCK1 expression. The results showed significantly higher expression of circDOCK1 in the BC tissues than in the non‐tumour tissues (Figure 1E). Furthermore, the qRT‐PCR analysis showed increased circDOCK1 expression in 3 BC cell lines (EJ‐M3, T24 and 5637) compared to that in the normal urothelial cell line SV‐HUC (Figure 1F). Localization of circDOCK1 detected by fluorescence in situ hybridization in EJ‐M3 cells showed that it was localized in the cytoplasm (Figure 1G). Taken together, these observations demonstrated that circDOCK1 was significantly upregulated in BC tissues and cell lines and exerted a potential oncogenic function in BC.

### 3.2 SicircDOCK1 inhibited the progression of BC in vitro

**Figure 2:**
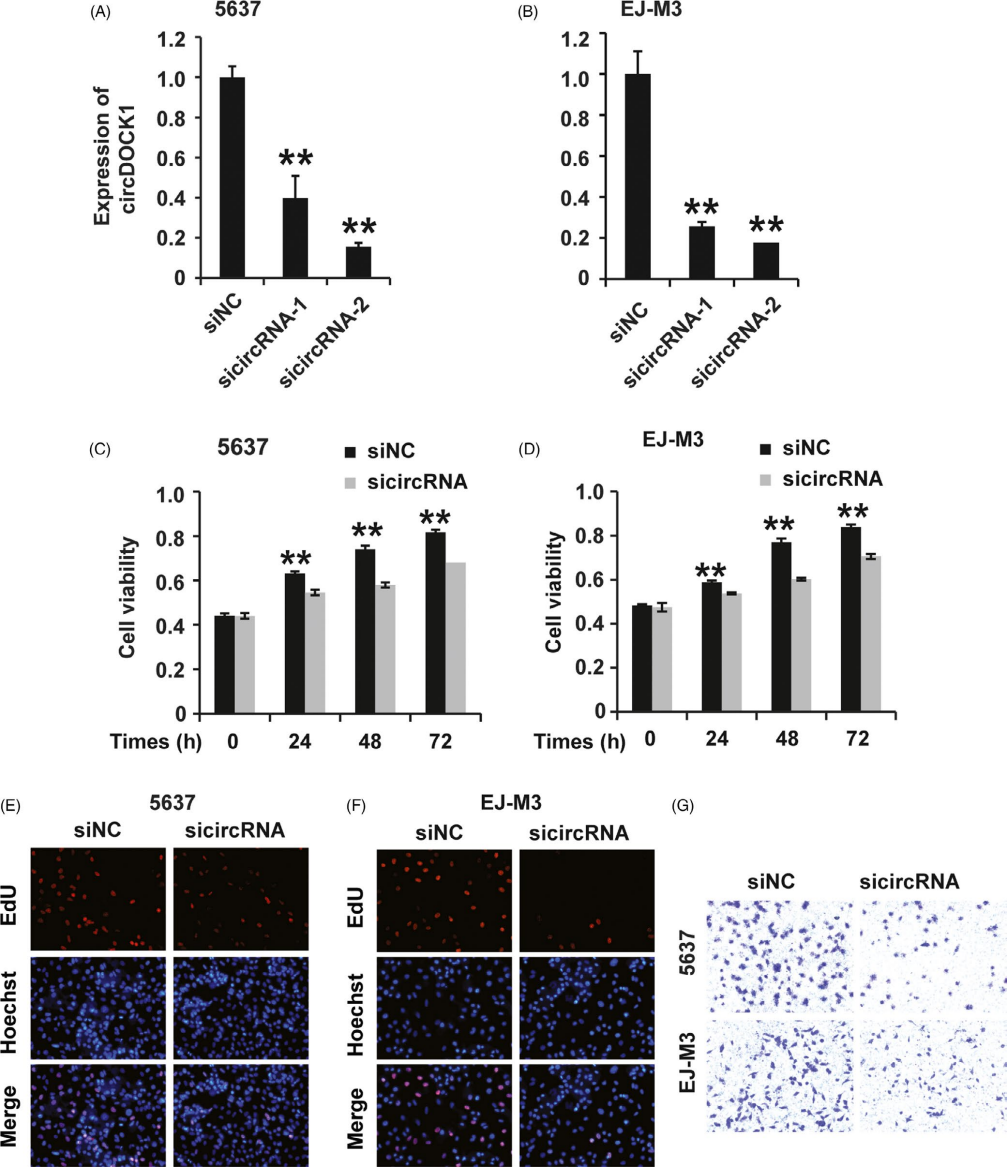
SicircDOCK1 inhibited the progression of BC cells in vitro. (A and B) qPCR analysis of the knockdown efficiency of siRNAs against circDOCK1 in the 5637 and EJ‐M3 cell lines. GAPDH was used as the control. (C and D) CCK8 assay for the viability of 5637 and EJ‐M3 cells transfected with the sicircDOCK1 and control siRNAs (siNC). (E and F) EdU assay for the proliferation of 5637 and EJ‐M3 cells transfected with the sicircDOCK1 and control siRNAs (siNC). (G) Transwell assay for the migration potential of 5637 and EJ‐M3 cells transfected with the sicircDOCK1 and control siRNAs (siNC). Each experiment was repeated a minimum of three times. The symbol * denotes a significant difference (*P* < 0.05), while ** represents a highly significant difference (*P* < 0.01) in a two‐tailed Student's *t* test

Given that circDOCK1 was significantly upregulated in BC tissues and cell lines, we hypothesized that circDOCK1 would play a certain role in BC. We then examined the function of circDOCK1 in the 5637 and EJ‐m3 BC cell lines. We first designed small interfering RNAs (siRNAs) specific to circDOCK1 and transfected them into 5637 and EJ‐m3 cells. qRT‐PCR analysis demonstrated that all three circDOCK1 siRNAs effectively inhibited the expression of circDOCK1 in both the 5637 and EJ‐m3 cell lines (Figure 2A,B); sicircDOCK1‐2 was used for the subsequent experiments. We then tested whether the inhibition of circDOCK1 could affect the progression of BC cells. The CCK8 assay showed that repression of circDOCK1 reduced the viability of 5637 and EJ‐m3 cells (Figure 2C,D). Furthermore, the ethynyl‐2ʹ‐deoxyuridine (EdU) assay showed that knockdown of circDOCK1 inhibited the proliferation of 5637 and EJ‐m3 cells (Figure 2E,F). In addition, the transwell assay demonstrated that the migration potential was decreased in 5637 and EJ‐m3 cells with circDOCK1 knockdown (Figure 2G). In summary, these results revealed that the inhibition of circDOCK1 suppressed the progression of BC.

### 3.3 CircDOCK1 acted as a molecular sponge for hsa‐miR‐132‐3p

**Figure 3:**
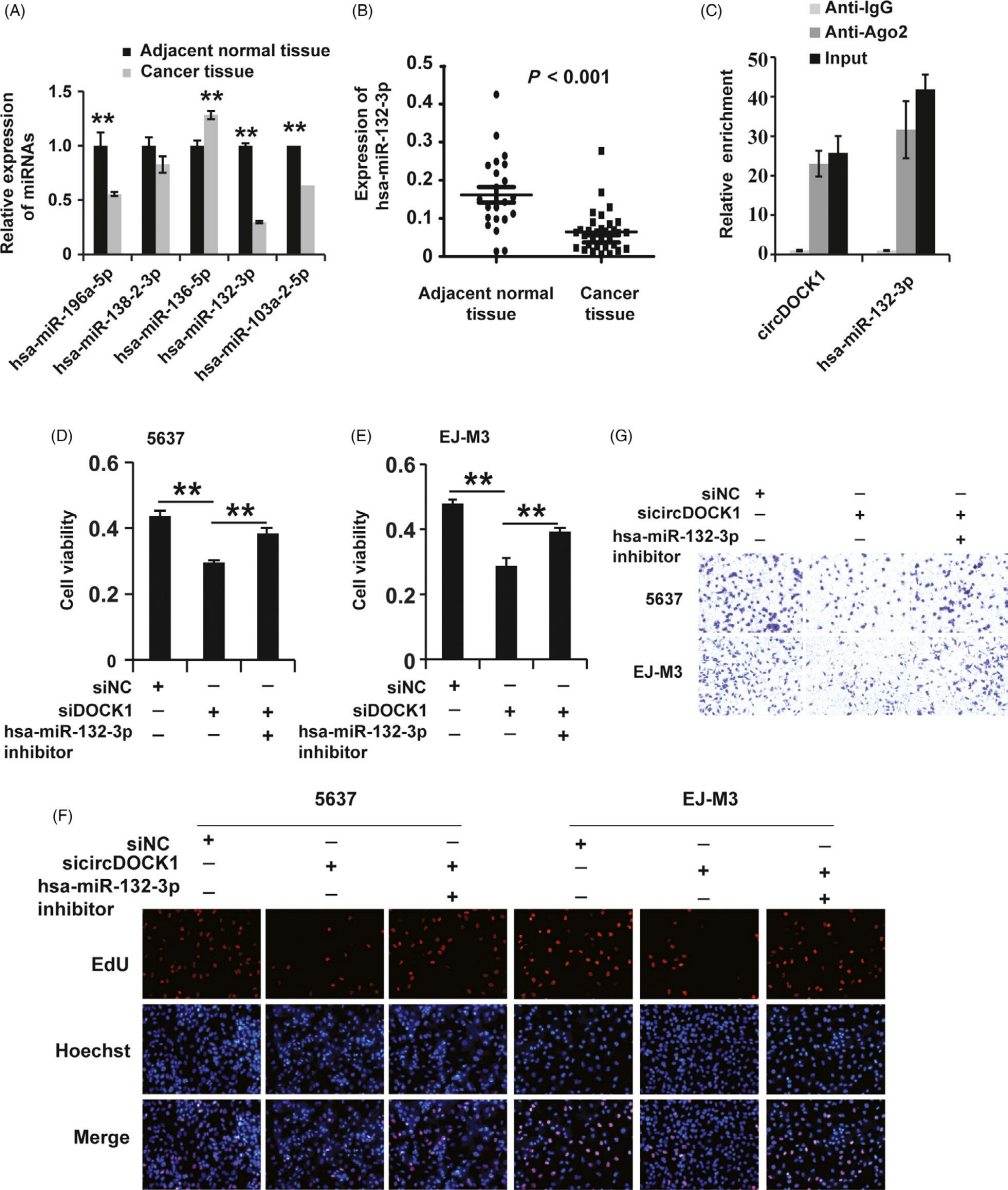
CircDOCK1 acted as a molecular sponge for hsa‐miR‐132‐3p. (A) qPCR analysis of the expression of candidate miRNA targets of circDOCK1 in BC and adjacent normal tissues. U6 was used as the control. (B) qPCR analysis of the expression of the candidate hsa‐miR‐132‐3p in BC (n = 23) and adjacent normal tissues (n = 32). U6 was used as the control. (C) The correlation between circDOCK1 and Ago2 was detected by a radioimmunoprecipitation (RIP) assay. Cell lysates were immunoprecipitated using an anti‐Ago2 antibody or IgG. The expression of circDOCK1 and hsa‐miR‐132‐3p was investigated by qRT‐PCR. (D and E) CCK8 assay for the viability of 5637 and EJ‐M3 cells transfected with the sicircDOCK1, sicircDOCK1 + miR‐132 inhibitor and control siRNAs (siNC). (F) EdU assay for the proliferation of 5637 and EJ‐M3 cells transfected with the sicircDOCK1, sicircDOCK1 + miR‐132 inhibitor and control siRNAs (siNC). (G) Transwell assay for the migration potential of 5637 and EJ‐M3 cells transfected with the sicircDOCK1, sicircDOCK1 + miR‐132 inhibitor and control siRNAs (siNC). Each experiment was repeated a minimum of three times. The symbol * denotes a significant difference (*P* < 0.05), while ** represents a highly significant difference (*P* < 0.01) in a two‐tailed Student's *t* test

Previous research has shown that circRNAs function mainly as miRNA sponges to bind functional miRNAs, leading to the regulation of gene expression.^20^, ^21^ We applied bioinformatic analysis to screen the miRNA binding sites on circDOCK1 and found that circDOCK1 possessed potential binding sites for hsa‐miR‐132‐3p, hsa‐miR‐196a‐5p, hsa‐miR‐138‐2‐3p, hsa‐miR‐136‐5p and hsa‐miR‐103a‐2‐5p. Then, we employed qPCR to detect the expression of these miRNAs in BC and normal tissues. We found that among the 5 candidate miRNAs, hsa‐miR‐132‐3p was the most significantly downregulated miRNA in BC tissues (Figure 3A). Then, we examined the expression of hsa‐miR‐132‐3p in numerous tissue samples and found that hsa‐miR‐132‐3p expression was lower in BC tissues than in non‐tumour tissues (Figure 3B). Subsequently, we applied a biotin‐coupled probe pull‐down assay to determine whether circDOCK1 could interact with hsa‐miR‐132‐3p. The results showed that circDOCK1 and hsa‐miR‐132‐3p were more abundant in the Ago2 pellet than in the control IgG pellet (Figure 3C). In summary, these observations suggested that circDOCK1 could sponge hsa‐miR‐132‐3p.

Then, we asked whether hsa‐miR‐132‐3p affects the role of circDOCK1 in BC. To answer this question, we treated BC cell lines with an hsa‐miR‐132‐3p inhibitor and subjected the cells to CCK8, EdU and transwell assays to examine the effects of hsa‐miR‐132‐3p on the role of circDOCK1. The CCK8 assay demonstrated that the suppression of hsa‐miR‐132‐3p by the hsa‐miR‐132‐3p inhibitor restored the sicircDOCK1‐induced reduction in viability in the 5637 and EJ‐m3 cell lines (Figure 3D,E). The EdU analysis showed that the hsa‐miR‐132‐3p inhibitor reverted the sicircDOCK1‐induced inhibition of proliferation in these cell lines (Figure 3F). In addition, the sicircDOCK1‐induced repression of the migration potential of 5637 and EJ‐m3 cells was also rescued by treatment with the hsa‐miR‐132‐3p inhibitor (Figure 3G). Taken together, these findings demonstrated that hsa‐miR‐132‐3p affected the role of circDOCK1 in BC cell lines.

### 3.4 Hsa‐miR‐132‐3p regulated the expression of Sox5 in BC

**Figure 4:**
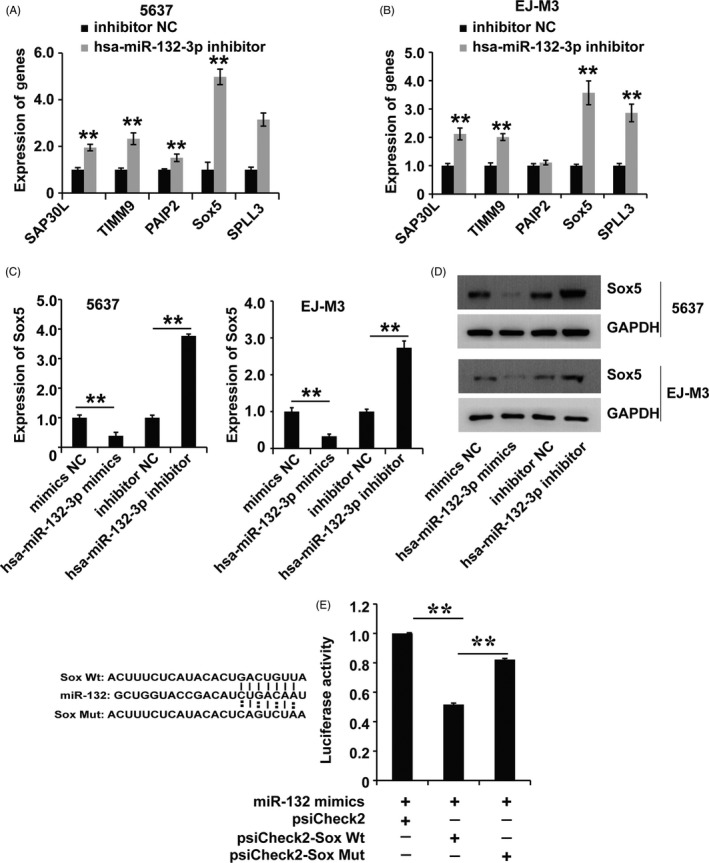
Hsa‐miR‐132‐3p regulated the expression of Sox5 in BC. (A and B) qPCR analysis of candidate mRNA targets of hsa‐miR‐132‐3p in 5637 and EJ‐M3 cells transfected with the miR‐132 inhibitor and control miRNAs (inhibitor NC). GAPDH was used as the control. (C and D) qPCR and Western blot analyses of Sox5 expression in 5637 and EJ‐M3 cells transfected with the miR‐132 inhibitor and control miRNAs (inhibitor NC). GAPDH was used as the control. (E) Luciferase reporter assay for the binding of hsa‐miR‐132‐3p to predict binding sites on Sox5. Each experiment was repeated a minimum of three times. The symbol * denotes a significant difference (*P* < 0.05), while ** represents a highly significant difference (*P* < 0.01) in a two‐tailed Student's *t* test

miRNAs can act by targeting mRNAs to regulate a variety of biological processes. Thus, we employed bioinformatics analysis with PicTar and TargetScan to screen the potential target mRNAs of hsa‐miR‐132‐3p. We found that several mRNAs, such as SAP30L, TIMM9, PAIP2, SPLL3 and Sox5, were potential targets of hsa‐miR‐132‐3p. To detect the target of hsa‐miR‐132‐3p in BC, we used qPCR to investigate the expression of the candidate mRNAs in both 5637 and EJ‐m3 cells transfected with the hsa‐miR‐132‐3p inhibitor and found that Sox5 was the mRNA with the greatest increase in expression in cells transfected with the hsa‐miR‐132‐3p inhibitor (Figure 4A,B). Then, we applied qRT‐PCR and Western blotting to determine the regulation of Sox5 by hsa‐miR‐132‐3p and found that hsa‐miR‐132‐3p downregulated the expression of Sox5 at the mRNA and protein levels in both EJ‐m3 and 5637 cells (Figure 4C,D). To further confirm the regulation of Sox5 expression by hsa‐miR‐132‐3p, we used a dual‐luciferase reporter assay to investigate the binding between hsa‐miR‐132‐3p and Sox5. The results demonstrated that the luciferase activity was decreased in cells co‐transfected with Sox5 and the hsa‐miR‐132‐3p mimic. However, the decrease was restored in cells co‐transfected with the hsa‐miR‐132‐3p mimic and a Sox5 mutant with the hsa‐miR‐132‐3p binding site mutated (Figure 4E). In summary, these observations revealed that hsa‐miR‐132‐3p regulated Sox5 expression in EJ‐m3 and 5637 cells.

### 3.5 Hsa‐miR‐132‐3p played a role via the regulation of Sox5 expression in BC

**Figure 5:**
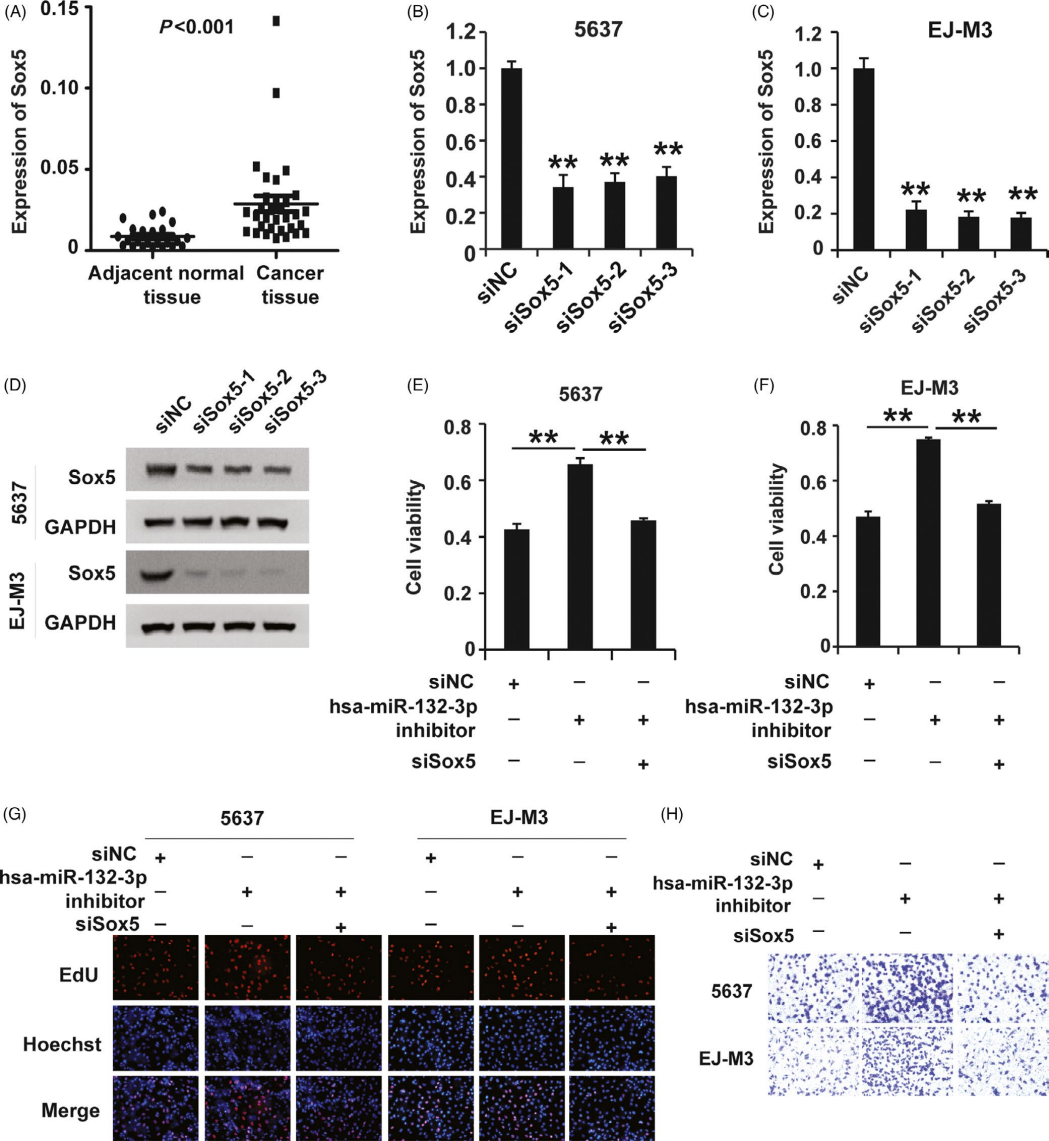
Hsa‐miR‐132‐3p plays a role in regulating Sox5 expression in BC. (A) qPCR analysis of Sox5 expression in BC (n = 23) and adjacent normal tissues (n = 32). GAPDH was used as the control. (B and C) qPCR analysis of the knockdown efficiency of siRNAs against Sox5 (siSox5) in 5637 and EJ‐M3 cells. GAPDH was used as the control. (D) Western blot analysis of the knockdown efficiency of siRNAs against Sox5 in 5637 and EJ‐M3 cells. GAPDH was used as the control. (E and F) CCK8 assay for the viability of 5637 and EJ‐M3 cells transfected with the miR‐132 inhibitor, miR‐132 inhibitor + siSox5 and control siRNAs (siNC). (G) EdU assay for the proliferation of 5637 and EJ‐M3 cells transfected with the miR‐132 inhibitor, miR‐132 inhibitor + siSox5 and control siRNAs (siNC). (H) Transwell assay for the migration potential of 5637 and EJ‐M3 cells transfected with the miR‐132 inhibitor, miR‐132 inhibitor + siSox5 and control siRNAs (siNC). Each experiment was repeated a minimum of three times. The symbol * denotes a significant difference (*P* < 0.05), while ** represents a highly significant difference (*P* < 0.01) in a two‐tailed Student's *t* test

Given that hsa‐miR‐132‐3p regulated the expression of Sox5, we asked whether Sox5 affected the role of hsa‐miR‐132‐3p in BC. To answer this question, we first examined the expression of Sox5 in BC tissues and cell lines. The qPCR analysis demonstrated that Sox5 expression was significantly higher in the BC tissues than in the non‐tumour tissues (Figure 5A). Next, we designed siRNAs specific to Sox5 and transfected them into 5637 and EJ‐m3 cells. qRT‐PCR and Western blotting demonstrated that all three Sox5 siRNAs (siSox5) effectively inhibited the expression of Sox5 in both 5637 and EJ‐m3 cells (Figure 5B‐D）; siSox5‐1 was chosen for the subsequent experiments. Then, we employed CCK8, EdU and transwell assays to examine the effect of Sox5 on the role of hsa‐miR‐132‐3p. The CCK8 assay showed that while the hsa‐miR‐132‐3p inhibitor induced an enhancement of cell viability in EJ‐m3 and 5637 cells, siSox5 inhibited this enhancement (Figure 5E,F). Furthermore, the EdU assay showed that siSox5 could rescue the hsa‐miR‐132‐3p inhibitor‐induced promotion of proliferation in BC cell lines (Figure 5G). In addition, the cell migration potential promoted by the hsa‐miR‐132‐3p inhibitor was also suppressed by Sox5 knockdown in BC cell lines. (Figure 5H). In summary, these observations demonstrated that hsa‐miR‐132‐3p exerted its effects via the regulation of Sox5 expression in BC.

### 3.6 CircDOCK1 regulated the progression of BC via the circDOCK1/hsa‐miR‐132‐3p/Sox5 pathway

**Figure 6:**
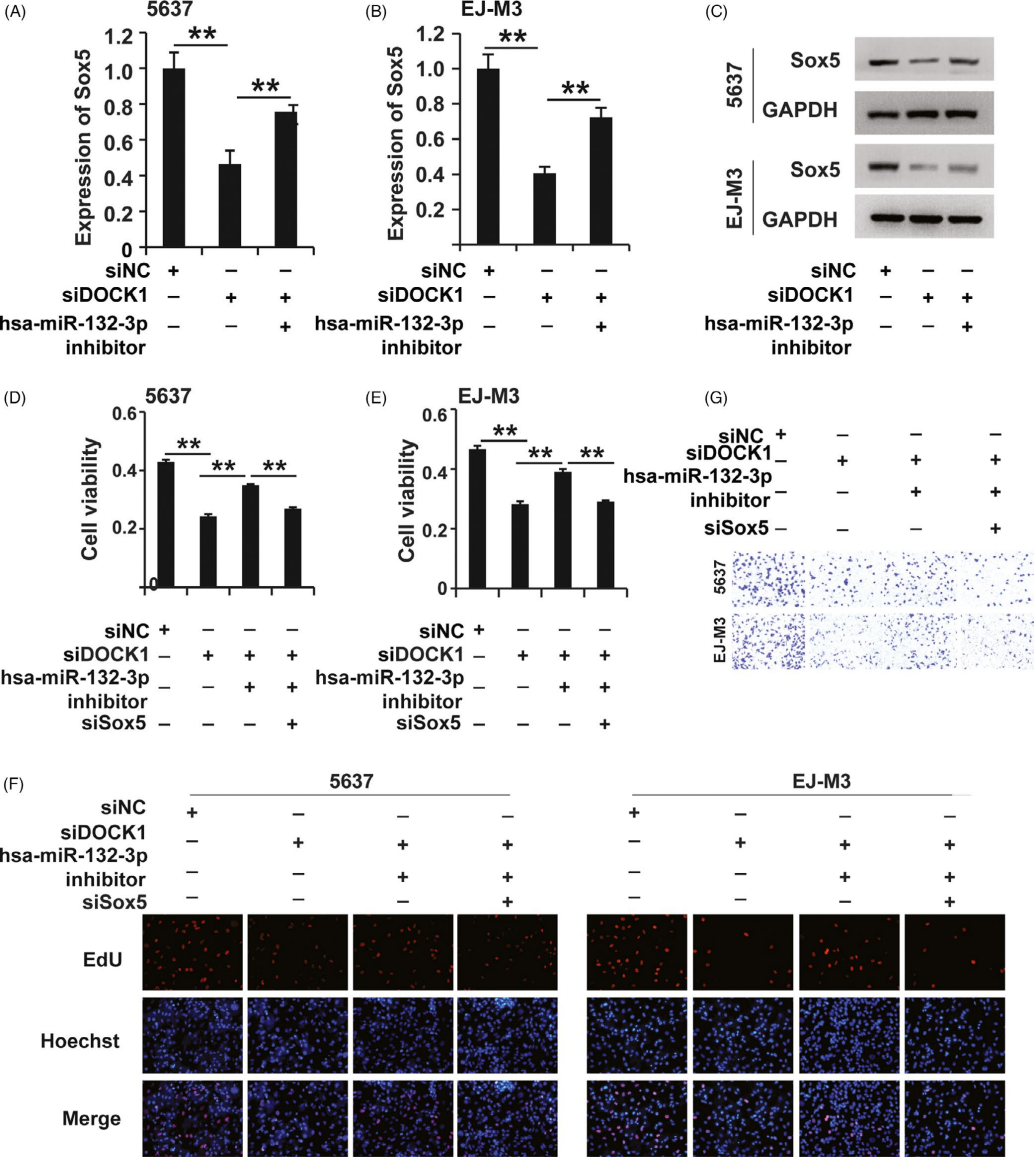
CircDOCK1 regulated the progression of BC via the circDOCK1/hsa‐miR‐132‐3p/Sox5 pathway. (A and B) qPCR analysis of Sox5 expression in 5637 and EJ‐M3 cells transfected with the sicircDOCK1 and control siRNAs (siNC). GAPDH was used as the control. (C) Western blot analysis of Sox5 expression in 5637 and EJ‐M3 cells transfected with the sicircDOCK1 and control siRNAs (siNC). GAPDH was used as the control. (D and E) CCK8 assays for the viability of 5637 and EJ‐M3 cells exposed to different treatments. (F) EdU assay for the proliferation of 5637 and EJ‐M3 cells exposed to different treatments. (G) Cell migration assay for the migration potential of 5637 and EJ‐M3 cells exposed to different treatments. Each experiment was repeated a minimum of three times. The symbol * denotes a significant difference (*P* < 0.05), while ** represents a highly significant difference (*P* < 0.01) in a two‐tailed Student's *t* test

As mentioned above, we showed that circDOCK1 sponged hsa‐miR‐132‐3p and that hsa‐miR‐132‐3p functioned through regulating Sox5 expression in BC. We thus hypothesized that circDOCK1 might exert its effects via the modulation of the circDOCK1/hsa‐miR‐132‐3p/Sox5 pathway in BC. To verify this hypothesis, we first used qPCR and Western blotting to determine whether circDOCK1 mediated the regulation of Sox5 by hsa‐miR‐132‐3p in BC. Interestingly, qPCR and Western blotting showed that sicircDOCK1 inhibited Sox5 expression and that the hsa‐miR‐132‐3p inhibitor restored that effect in both the 5637 and EJ‐m3 cell lines (Figure 6A‐E). Then, we sought to examine the function of circDOCK1/hsa‐miR‐132‐3p/Sox5 pathway modulation in BC. The CCK8, EdU and transwell assays demonstrated that the modulation of the circDOCK1/hsa‐miR‐132‐3p/Sox5 pathway regulated cell viability, cell proliferation and cell migration potential in both the 5637 and EJ‐m3 cell lines (Figure 6F,G). Taken together, these results demonstrated that circDOCK1 regulated the progression of BC via the circDOCK1/hsa‐miR‐132‐3p/Sox5 pathway.

### 3.7 Inhibition of circDOCK1 repressed the growth of xenograft tumours in vivo

**Figure 7:**
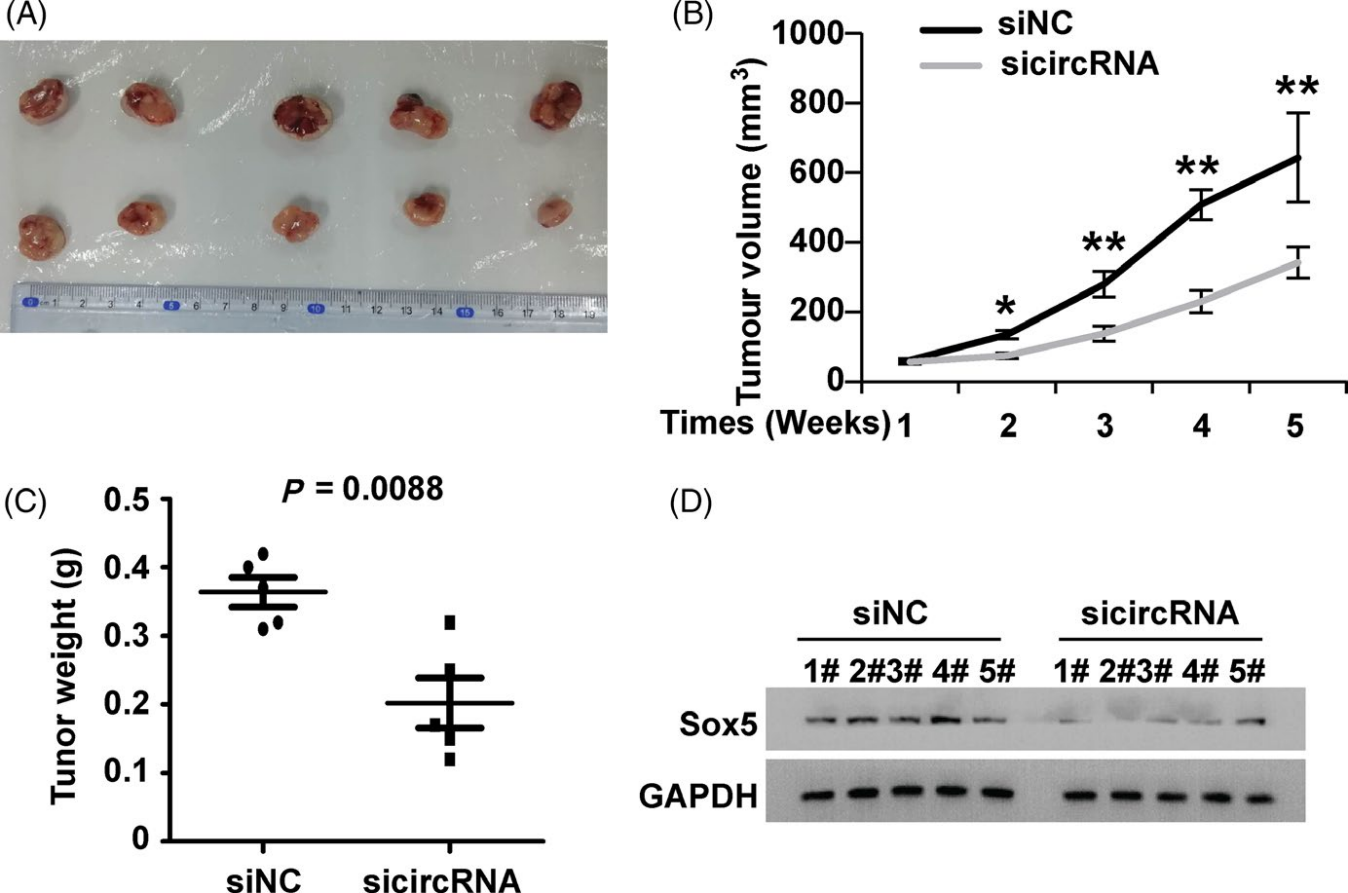
Inhibition of circDOCK1 repressed the growth of xenograft tumours in vivo. (A) Representative images of the xenograft tumours in BALB/c nude mice. (B) The growth curves of xenograft tumours at different time points (wk). (C) The relative weights of tumours were analysed at different time points (wk). (D) Western blot analysis of Sox5 expression in xenograft tumours and tissues of BALB/c nude mice. The symbol * denotes a significant difference (*P* < 0.05), while ** represents a highly significant difference (*P* < 0.01) in a two‐tailed Student's *t* test

As we indicated above, circDOCK1 affected the progression of BC in vitro. To determine whether circDOCK1 affected tumour growth in vivo, EJ‐m3 cells with circDOCK1 inhibition were injected subcutaneously into nude mice (Figure 7A). Then, the tumour volumes were measured weekly after injection. We found that the tumour volumes in the circDOCK1 inhibition group were significantly lower than those in the control group (*P* < 0.05, Figure 7B). A similar phenomenon was observed for the average tumour weights (*P* < 0.01, Figure 7C). In addition, the Western blot analysis demonstrated that the expression of Sox5 was downregulated in the tumours with circDOCK1 inhibition (Figure 7D). In summary, these observations showed that circDOCK1 repressed the growth of xenograft tumours in vivo.

## 4 DISCUSSION

In recent years, an increasing number of studies have indicated the existence and function of circRNAs, which form closed‐loop structures without a 5′ cap or 3′ tail.^15^, ^23^, ^24^ Although once considered noise and transcription artefacts, circRNAs are abundant, conserved, stable and cell type‐specific, and they exert important functions in various cellular processes and in the development and progression of human physiological and pathological processes.^25^, ^26^ Previous studies have demonstrated that circRNAs play important roles in numerous human carcinomas, including lung carcinoma, hepatocellular carcinomas, osteosarcoma, ovarian carcinoma, prostate carcinoma, colon carcinoma, pancreatic carcinoma, glioma, gastric carcinoma, breast carcinoma and BC.^29^, ^30^ However, to date, only a few circRNAs have been identified and examined. In this study, we systematically identified the differential expression of circRNAs in BC tissues using a high‐throughput microarray and found that the expression of 734 circRNAs, including 478 upregulated and 256 downregulated circRNAs, was significantly altered with a fold change of >2.0 and a *P* of <0.05.

As one of the most commonly diagnosed urologic carcinomas with distinctive morbidity and mortality in males worldwide, BC inflicts enormous physical, psychological and economic burdens on patients and their families, and it also imposes a heavy social burden. Previous research has summarized the hallmarks of carcinomas as follows: the presence of self‐sufficient growth signals, an insensitivity to growth signals, resistance to cell death, an unlimited ability to replicate, sustained angiogenesis, tissue infiltration and metastasis, avoidance of immune destruction, the promotion of tumour inflammation, abnormalities in cellular metabolism, and genomic instability and mutation.^32^, ^33^ Thus, the aim of carcinoma therapy is to reverse or inhibit these hallmarks of carcinomas. However, the characteristics and underlying mechanism of the pathological occurrence and development of tumours are complicated and remain largely unclear. Studies have documented that circRNAs play key roles in various cellular processes.^23^ In our study, we found that circDOCK1 was upregulated in BC cell lines and that circDOCK1 knockdown reduced cell viability, inhibited cell proliferation and reduced the cell migration potential in both the EJ‐m3 and 5637 cell lines. Thus, our study confirmed the role of this circRNA in BC. However, the overall role and underlying mechanism of circDOCK1 in BC need to be revealed.

CircRNAs could play a role via ceRNA hypothesis by acting as miRNA sponges, regulating parental gene transcription and interactions with RNA‐binding proteins.^9^, ^34^, ^35^ The ceRNA hypothesis defined that lncRNAs could act as molecular sponges that compete with mRNAs for binding to microRNAs, leading to the inhibition of the activities of miRNAs and thus releasing the repression of mRNAs.^17^ As a new member of the ceRNA family, circRNAs have been documented to affect the development and progression of human disease via acting as ceRNAs.^36^, ^37^ Chengdi Yang and colleagues revealed that circRNA circ‐ITCH inhibited BC progression by sponging miR‐17/miR‐224 and regulating p21 and PTEN expression.^38^ Here, we found that circDOCK1 exerted its function by sponging hsa‐miR‐132‐3p, leading to the regulation of Sox5 expression and thus forming the circDOCK1/hsa‐miR‐132‐3p/Sox5 regulatory axis. Furthermore, modulation of the circDOCK1/hsa‐miR‐132‐3p/Sox5 regulatory axis in BC progression was approved.

Currently, biomarkers specific for most carcinomas are still largely undefined. The expression of circRNAs has been reported to differ significantly between tumour tissues and the adjacent normal tissues in numerous carcinomas; thus, circRNAs specifically expressed in a variety of solid tumours are potential novel biomarkers for tumour diagnosis and prognosis.^39^, ^40^ Here, we have examined the overall expression and function of the circDOCK1/hsa‐miR‐132‐3p/Sox5 pathway in BC, and propose that the expression profile of players in the circDOCK1/hsa‐miR‐132‐3p/Sox5 regulatory axis might be a biomarker for the assessment of BC risk and that these molecules might be novel therapeutic targets in BC.

## CONFLICTS OF INTEREST

The authors declare no competing financial interests.
